# Cross-Talk Immunity of PEDOT:PSS Pressure Sensing Arrays with Gold Nanoparticle Incorporation

**DOI:** 10.1038/s41598-017-12420-5

**Published:** 2017-09-25

**Authors:** Rajat Subhra Karmakar, Yu-Jen Lu, Yi Fu, Kuo-Chen Wei, Shun-Hsiang Chan, Ming-Chung Wu, Jyh-Wei Lee, Tzu-Kang Lin, Jer-Chyi Wang

**Affiliations:** 1 Department of Electronic Engineering, Chang Gung University, Guishan Dist., 33302 Taoyuan, Taiwan; 2 Department of Neurosurgery, Chang Gung Memorial Hospital, Guishan Dist., 33305 Taoyuan, Taiwan; 3 School of Traditional Chinese Medicine, Chang Gung University, Guishan Dist., 33302 Taoyuan, Taiwan; 4 School of Medicine, Chang Gung University, Guishan Dist., 33302 Taoyuan, Taiwan; 5 Department of Chemical and Materials Engineering, Chang Gung University, Guishan Dist., 33302 Taoyuan, Taiwan; 6 Department of Materials Engineering, Ming Chi University of Technology, Taishan Dist., 24301 New Taipei City, Taiwan; 7 Center for Thin Films Technologies and Applications, Ming Chi University of Technology, Taishan Dist., 24301 New Taipei City, Taiwan; 8 College of Engineering, Chang Gung University, Guishan Dist., 33302 Taoyuan, Taiwan; 9 Department of Electronic Engineering, Ming Chi University of Technology, Taishan Dist., 24301 New Taipei City, Taiwan

## Abstract

In this study, the cross-talk effects and the basic piezoresistive characteristics of gold nanoparticle (Au-NP) incorporated poly(3,4-ethylenedioxythiophene):poly(styrenesulfonate) (PEDOT:PSS) pressure sensing 2 × 2 arrays are investigated using a cross-point electrode (CPE) structure. Transmission electron microscopy (TEM), scanning electron microscopy (SEM), and energy-dispersive X-ray spectroscopy (EDS) mappings were carried out to confirm the incorporation of Au-NPs in the PEDOT:PSS films. A solution mixing process was employed to incorporate the nanoparticles. When the diameter of the Au-NPs incorporated in the PEDOT:PSS films (Au-NPs/PEDOT:PSS) was 20 nm, the piezoresistive pressure sensing 2 × 2 arrays were almost immune to cross-talk effects, which enhances the pressure sensing accuracy of the array. The Au-NPs render the PEDOT:PSS films more resilient. This is confirmed by the high plastic resistance values using a nanoindenter, which reduce the interference between the active and passive cells. When the size of the Au-NPs is more than 20 nm, a significant cross-talk effect is observed in the pressure sensing arrays as a result of the high conductivity of the Au-NPs/PEDOT:PSS films with large Au-NPs. With the incorporation of optimally sized Au-NPs, the PEDOT:PSS piezoresistive pressure sensing arrays can be promising candidates for future high-resolution fingerprint identification system with multiple-electrode array structures.

## Introduction

Nanomaterials are crucial elements of many recent technological and industrial advances including electronics^1–3^, fuel cells^4^, batteries^5^, agriculture^6^, food^7^, and medicine^8,9^. An extensive range of organic and inorganic nanomaterials has been investigated in the recent past. Among these nano-structured materials, metal nanoparticles are particularly noteworthy because of their easy synthesis, tunable properties, and high surface to volume ratio. Of the different types of metallic nanoparticles, gold nanoparticles (Au-NPs) have been intensely researched upon the scientific community. Their ease of synthesis and unique properties make Au-NPs ideal candidates for use as conductors for printable inks and electronic chips, photodynamic therapy, therapeutic agent delivery, and optical, chemical, or bio-sensing applications^10^. In addition to these standalone applications, Au-NPs are also used for pressure sensing due to their wide and diverse range of implementation as conductive fillers in pressure sensing membranes^11–15^.

Pressure sensors are very important for industrial equipment and are extensively used for control and monitoring in thousands of applications in the biomedical, environmental, space, and automotive fields^16–19^. There are three predominant types of pressure sensors in the market – capacitive, piezoelectric, and piezoresistive pressure sensors. Piezoresistive pressure sensors are the most commonly employed, owing to their high sensitivity and low cost. The materials generally used for piezoresistive pressure sensors are silicon, polysilicon thin films, bonded metal foils, sputtered thin films, and inkjet-printed films^20–22^. Recently, metal particle embedded films, especially those featuring Au-NPs, have been used for piezoresistive pressure sensing applications and many efforts have been undertaken to further such utilization. For example, Stassi *et al*. reported the embedding of three morphologically different metal conductive “spiky” particles, including gold nanostars, in a silicone-based polymeric matrix for piezoresistive composites based on a tunneling conduction mechanism^11,12^. A new strategy to design mechanical sensors composed of Au-NP arrays on an elastomeric substrate of polydimethylsiloxane (PDMS) and based on metal-enhanced fluorescence phenomena was demonstrated^13^. For the wearable pressure sensor application, a gold nanowire-impregnated tissue paper could be sandwiched between blank and patterned PDMS sheets with interdigitated electrode arrays^14^. It is also reported that resistive pressure gauges based on a 1,6-hexanedithiol (DMH) cross-linked Au-NP membrane can serve as strain sensitive transducers^15^. However, the cross-talk effects in piezoresistive pressure sensing arrays incorporated with nanoparticles have not yet been investigated.

In this work, spherical Au-NPs of different diameters have been incorporated into poly (3,4-ethylenedioxythiophene):polystyrene sulfonate (PEDOT:PSS) films for the elimination of cross-talk effects in piezoresistive pressure sensing 2 × 2 arrays. PEDOT:PSS is a conductive polymer widely used in modern electronics due to its high electrochemical and thermal stability, high conductivity, and good optical properties and transparency^23,24^. It also possesses impressive piezoresistive characteristics, which make it an excellent candidate for pressure sensing applications^24–28^. The cross-point electrode (CPE) array structure, common in emerging memory devices such as the resistive random access memory (RRAM) devices^29,30^, is being utilized for future high-density strain sensor arrays, where the number of active cells can be increased by increasing the number of top and bottom electrodes^31,32^. Unfortunately, cross-talk effects, which result from the interference between adjacent pressure-sensing cells, create measurement noise and degrade the sensing behavior of the pressure sensing arrays. To eliminate these cross-talk effects, Au-NPs are incorporated into the PEDOT:PSS films (Au-NPs/PEDOT:PSS) and the diameter-dependent piezoresistive and interference characteristics are investigated. On the basis of the material, electrical, and simulation analyses, a theoretical model is proposed to explain the elimination of cross-talk effects in the Au-NPs/PEDOT:PSS pressure sensing arrays. Such systems can potentially be used to fabricate future high-resolution multiple-electrode array structures.

## Results

### Material Analysis

Conductive nanocomposite films consisting of PEDOT:PSS and Au-NPs of six different sizes were prepared using a solution mixing process. The Au-NPs used in this study are denoted as 5, 10, 20, 30, 50, and 80 nm, and are individually mixed with PEDOT:PSS to obtain the nanocomposite films with superior piezoresistive and cross-talk characteristics. The transmission electron microscopy (TEM) images of the Au-NPs with different diameters are shown in Fig. S1. The average diameters of the Au-NPs used in this study are as follows –5.0 nm ± 0.6 nm (5 nm), 9.8 nm ± 1.2 nm (10 nm), 20.2 nm ± 3.2 nm (20 nm), 33.3 nm ± 4.6 nm (30 nm), 54.0 nm ± 5.4 nm (50 nm), and 85.2 nm ± 9.3 nm (80 nm). The PEDOT:PSS films incorporated with Au-NPs were spin-coated on indium tin oxide/polyethylene terephthalate (ITO/PET) substrates to obtain the Au-NPs/PEDOT:PSS films with a thickness of 2.5 μm. The chemical composition of the Au-NPs/PEDOT:PSS films was analyzed by energy-dispersive X-ray spectroscopy (EDS) and the results are listed in Supplementary Table S1. The average Au/S ratios of the Au-NPs/PEDOT:PSS films increase with an increase in the Au-NP diameters. The Au-NPs were well dispersed in the PEDOT:PSS matrices, as observed from the EDS mapping of various Au-NPs/PEDOT:PSS films (Supplementary Fig. S2). Hence, we employed these PEDOT:PSS films incorporated with Au-NPs of different sizes to further investigate their piezoresistive and cross-talk behaviors.

### Piezoresistive Sensitivity and Cross-Talk Effects

**Figure 1:**
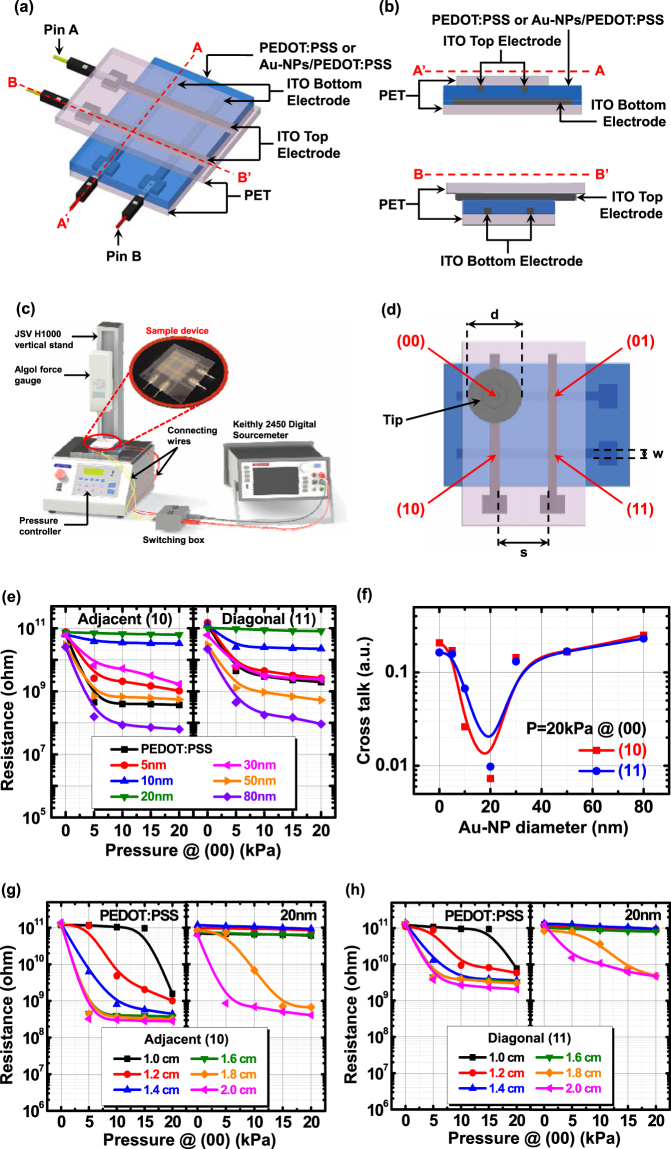
(**a**) Schematic diagram of the Au-NP incorporated PEDOT:PSS pressure sensing 2 × 2 arrays and (**b**) the cross-sectional structures in A-A’ and B-B’ direction. (**c**) The measurement setup of the pressure sensing arrays. (**d**) The top view of a 2 × 2 CPE pressure sensing array structure with an active cell (00), adjacent cells (01, 10) and diagonal cell (11). The electrode width (w) is 0.3 cm, the spacing between two electrodes (s) is 0.7 cm and the tip size (**d**) is in the range of 1 to 2 cm. (**e**) The cross-talk effects of the PEDOT:PSS pressure sensing 2 × 2 arrays with different sizes of Au-NP incorporation. The tip size used in this study is 1.6 cm. The pressure is applied at active (00) cell and the resistance values are measured at adjacent (10) and diagonal (11) cells. The cross-talk values (*CT*) are calculated and shown in (**f**). The tip size dependence on cross-talk effects of the PEDOT:PSS pressure sensing 2 × 2 arrays without and with Au-NP incorporation of 20 nm in diameter for (**g**) the adjacent (10) and (**h**) diagonal (11) cells. The tip sizes used in this study are in the range of 1 to 2 cm.

Before examining the cross-talk effects of the piezoresistive pressure sensing 2 × 2 arrays with Au-NPs/PEDOT:PSS films, the typical piezoresistive characteristics of the sensors were investigated. The schematic diagram in Supplementary Fig. S3 shows a 1 × 1 CPE structure of the sensors with Au-NPs/PEDOT:PSS films. The piezoresistive and reversible testing characteristics of the pressure sensors are shown in Supplementary Figs S4 and S5, respectively. To obtain the piezoresistive sensitivity of these samples, the slopes of the curves in Supplementary Fig. S4a at a pressure lower than 5 kPa, which is the threshold point between the fast-response stage and the near-saturation stage^33^, are calculated according to equation (1) and the results are displayed in Supplementary Fig. S4b ^27^.

1 $$S=\frac{\mathrm{log}({R}_{0})-\,\mathrm{log}({R}_{{\rm{5kpa}}})}{{\rm{5kpa}}}$$

where *S* is the piezoresistive sensitivity, *R*_0_ is the resistance when no pressure is applied, and *R*_5kPa_ is the resistance when a pressure of 5 kPa is applied. When the size of the Au-NPs increases, the piezoresistive sensitivity of the pressure sensors initially increases and then decreases. The optimized sensitivity of the Au-NPs/PEDOT:PSS pressure sensors with Au-NPs of 20 nm in diameter is 0.596 kPa^−1^. This is due to the dramatic decrease in *R*_0_ for those sensors incorporated with Au-NPs of more than 20 nm in diameter. Furthermore, the pressure sensors fabricated with Au-NPs/PEDOT:PSS films exhibit an excellent reversible testing characteristic for 5 cycles, thus providing a faster response with a shorter relaxation time (Supplementary Fig. S5), as compared to those with PEDOT:PSS films. The schematic diagram and cross-sectional views in the A-A′ and B-B′ directions of the piezoresistive pressure sensing arrays with a 2 × 2 CPE structure are shown in Fig. 1a,b, respectively. The flow chart for the measurement algorithm used to measure the cross-talk effects in the pressure sensing 2 × 2 arrays is illustrated in Supplementary Fig. S6 and the measurement system was set up as shown in Fig. 1c. An aluminum tip is attached to an ALGOL force gauge through which the pressure is applied. The pressure is applied at the active (00) cell and the resistances of the adjacent (10) and diagonal (11) cells are measured (Fig. 1d). A tip with a diameter (d) of 1.6 cm was used in this study. The electrode width (w) was 0.3 cm and the spacing between the two electrodes (s) was 0.7 cm. The cross-talk effects of the piezoresistive pressure sensing arrays fabricated with the Au-NPs/PEDOT:PSS films are graphically illustrated in Fig. 1e. The cross-talk value (*CT*_(i j)_) of the adjacent (10) and diagonal (11) cells for the pressure applied at the active (00) cell were extracted using the following equation

2 $$C{T}_{({\rm{i}}{\rm{j}})}=|\frac{\mathrm{log}({R}_{({\rm{i}}{\rm{j}})\_\mathrm{20kpa}})-\,\mathrm{log}({R}_{({\rm{i}}{\rm{j}})\_0})}{\mathrm{log}({R}_{({\rm{i}}j\_0)})}|$$

where *CT*_(i j)_ is the value of the cross-talk of the adjacent (10) or diagonal (11) cells, *R*_(i j)_0_ is the resistance at the adjacent (10) or diagonal (11) cells when no pressure is applied at the active (00) cell, and *R*_(i j)_20kPa_ is the resistance at the adjacent (10) or diagonal (11) cells when a pressure of 20 kPa is applied at the active (00) cell. The results are shown in Fig. 1f. In this figure, we can see that for the adjacent (10) cell, the pressure sensing arrays with the PEDOT:PSS film shows a significant change in resistance when the pressure is applied at the active (00) cell, similar to the basic piezoresistive characteristics of pressure sensors (Supplementary Fig. S4a). The cross-talk effect is prominent in the pressure sensing arrays with the PEDOT:PSS film, which causes a severe interference between the active and passive cells. A similar behavior is observed for the diagonal (11) cell of the pressure sensing arrays with the PEDOT:PSS film (Fig. 1e). To reduce the cross-talk effects, Au-NPs were incorporated in the PEDOT:PSS films and an improvement in both the adjacent (10) and diagonal (11) cells is immediately obvious. It is important to note that the Au-NPs/PEDOT:PSS pressure sensing arrays with 20 nm Au-NPs are nearly immune to cross-talk effects, i.e. negligible *CT* values of 0.0073 and 0.0098 for the adjacent and diagonal cells, respectively, are obtained. However, in the Au-NPs/PEDOT:PSS pressure sensing arrays with Au-NPs larger than 20 nm in diameter, the cross-talk effects of both the adjacent (10) and diagonal (11) cells become significant.

To further investigate the cross-talk behavior of the pressure sensing arrays fabricated with the Au-NPs/PEDOT:PSS films, aluminum tips of different sizes were used for measurement. Six tips with diameters in the range of 1 to 2 cm were used to vary the active area subjected to pressure. The area-dependent cross-talk effects of the adjacent (10) and diagonal (11) cells of the PEDOT:PSS and Au-NPs/PEDOT:PSS pressure sensing arrays with 20 nm Au-NPs are shown in Fig. 1g,h, respectively. In the pressure sensing arrays with the PEDOT:PSS film, the cross-talk effect is extremely severe, even when the tip was only 1 cm. On the other hand, the Au-NPs/PEDOT:PSS pressure sensing arrays with 20 nm Au-NPs are almost immune to cross-talk effects in both the adjacent (10) and diagonal (11) cells, until a tip diameter of 1.6 cm is reached. At tip diameters of more than 1.8 cm (because the tip covers the neighboring electrodes), both the adjacent (10) and diagonal (11) cells will be influenced by the pressure applied at the active (00) cell, leading to an obvious decrease in the resistance.

### Mechanical Properties

**Figure 2:**
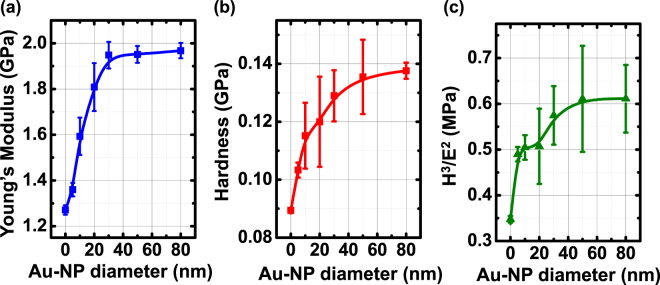
(**a**) Young’s modulus, (**b**) hardness, and (**c**) plastic resistance parameter (*H* ^3^/*E* ^2^) of the PEDOT:PSS films with Au-NP incorporation. The measurement was performed by the nanoindenter system.

The mechanical properties of the PEDOT:PSS films incorporated with Au-NPs of different diameters were studied to explain the elimination of cross-talk effects in the pressure sensing arrays. All the samples were spin-coated at 500-rpm to obtain an approximately 2.5 µm thick film. The Young’s modulus or elastic modulus (*E*) of the films, was calculated and expressed as follows^34^

3 $$\frac{1}{{E}_{r}}=\frac{1-{v}^{2}}{E}+\frac{v{i}^{2}}{Ei}$$

where *E*_r_ and *ν* are the reduced elastic modulus and Poisson’s ratio, respectively, of the Au-NPs/PEDOT:PSS nanocomposite films. Here, the experimental value of *ν* has been set at 0.35^35^. On the other hand, *E*_i_ and *ν*_i_ are attributes of the diamond indenter and their values are 1141 GPa and 0.07, respectively. The hardness (*H*) of the nanocomposite films was calculated using the following equation

4 $$H=\frac{{P}_{\max }}{{A}_{p}}$$

where *P*_max_ is the maximum applied indentation load and *A*_p_ is the projected area of contact. It can be found that the Young’s modulus increases significantly with an increase in the size of the Au-NPs (Fig. 2a), implying that the nanocomposite films have become more rigid. The Young’s modulus of the Au-NPs/PEDOT:PSS films with 80 nm Au-NPs is 1.97 GPa, which is quite close to the Young’s modulus of the pure Au-NPs (2 GPa), as reported by Schlicke *et al*.^36^. The same trend can be observed in the hardness values (Fig. 2b); this can be ascribed to the enhancement in the stiffness of the PEDOT:PSS films upon the incorporation of Au-NPs. The change in *H* ^3^/*E* ^2^ of the Au-NPs/PEDOT:PSS films is presented as a function of the Au-NP particle size (Fig. 2c). This parameter (denoted as the plastic resistance parameter) can be derived as^37^.

5 $${P}_{y}=0.78{r}^{2}\frac{{H}^{3}}{{E}^{2}}$$

where *H* is the hardness, *E* is the Young’s modulus, and *P*_y_ is the applied load needed to initiate plastic deformation when a rigid sphere of radius *r* is brought into contact with an elastic/plastic half space^34^. This equation is derived from Johnson’s analysis^38^ based on the fact that the hardness of a material can be estimated as approximately 3 times its yield strength^37,39^. It shows that the applied pressure must be high to induce plasticity in materials with large plastic resistance parameter values (i.e. *H* ^3^/*E* ^2^). We can observe that the *H* ^3^/*E* ^2^ values of the Au-NPs/PEDOT:PSS films increase with an increase in the Au-NP size (Fig. 2c). On the basis of the data and observations presented above, it can be deduced that the high Young’s modulus and hardness of the Au-NPs/PEDOT:PSS films make the fabricated pressure sensing arrays difficult to deform under pressure, thus leading to the elimination of cross-talk effects (Fig. 1e). The typical piezoresistive, cross-talk, and mechanical properties of the PEDOT:PSS pressure sensing arrays with various sizes of Au-NP incorporation are summarized in Table 1.

**Table 1:** Summary of the typical piezoresistive, cross-talk and mechanical properties of the PEDOT:PSS pressure sensing arrays with various sizes of Au-NP incorporation.

Data	1 × 1 pressure sensor		2 × 2 pressure sensing array		Mechanical properties		
	Sensitivity (kPa^−1^)	Relaxation time (sec)	Cross-talk (adjacent)	Cross-talk (diagonal)	Young’s Modulus (E) (GPa)	Hardness (H) (GPa)	H^3^/E^2^ (MPa)
**Sample**							
PEDOT:PSS	0.551	5	0.207	0.164	1.271	0.089	0.347
5 nm Au-NPs	0.59	3.2	0.171	0.157	1.36	0.103	0.49
10 nm Au-NPs	0.594	3	0.026	0.067	1.593	0.115	0.505
20 nm Au-NPs	0.596	2.7	0.0073	0.0098	1.808	0.12	0.507
30 nm Au-NPs	0.58	2.2	0.144	0.131	1.948	0.129	0.575
50 nm Au-NPs	0.535	2.1	0.166	0.168	1.951	0.135	0.611
80 nm Au-NPs	0.511	2.3	0.251	0.23	1.968	0.138	0.612

## Discussion

**Figure 3:**
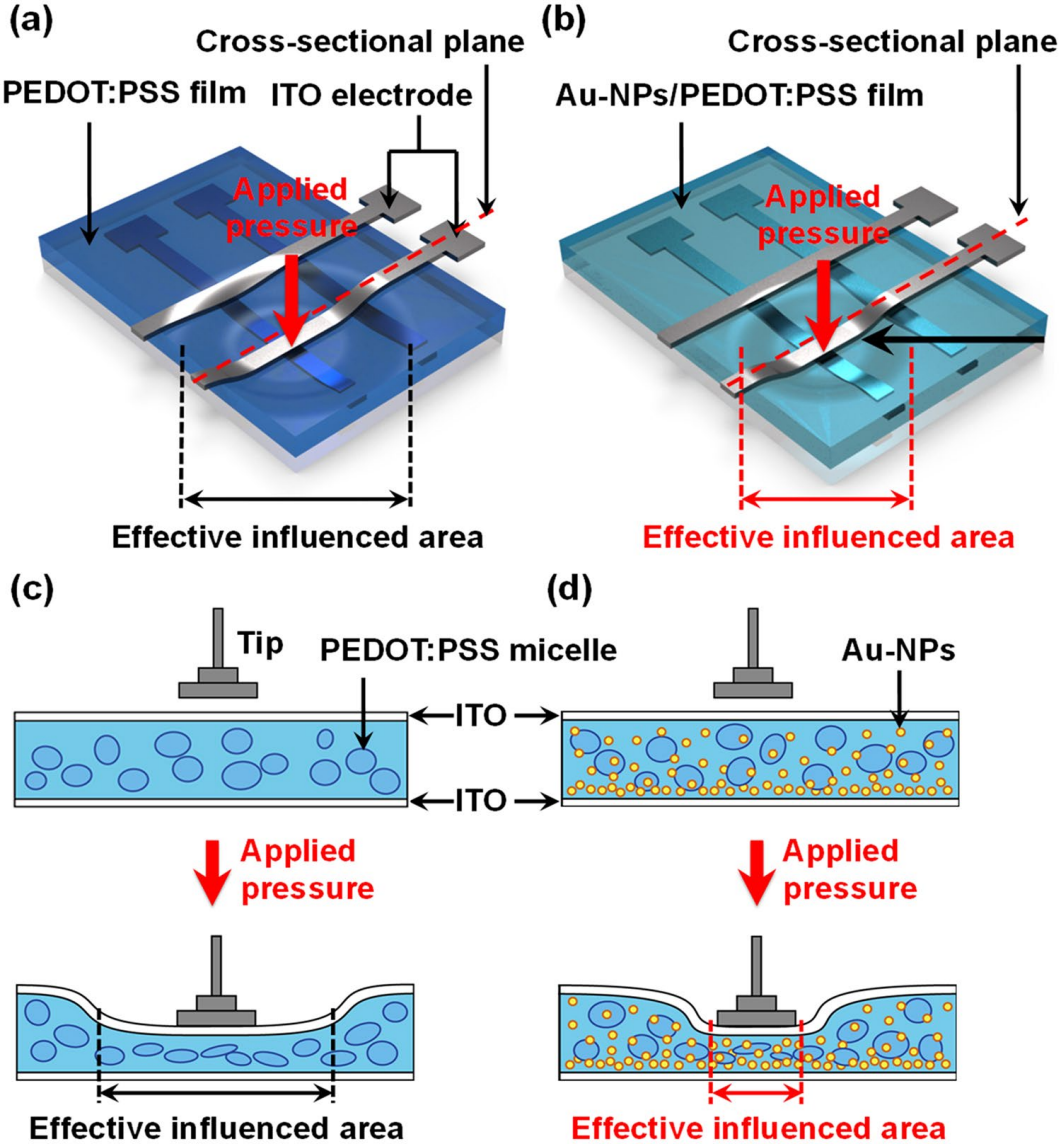
3D film deformed schematics of the PEDOT:PSS pressure sensing 2 × 2 arrays (**a**) without and (**b**) with Au-NP incorporation. The corresponding cross-sectional structure of the 3D schematics is shown in (**c**,**d**) respectively. The effective influenced area is defined as the particular area of the nanocomposite films which are influenced by the applied pressure.

The 3D schematics of the film deformation of piezoresistive pressure sensing arrays with PEDOT:PSS and Au-NPs/ PEDOT:PSS films are used to show the characteristics of the cross-talk effects (Fig. 3). As discussed in the previous section, cross-talk effects arise from the interference of an active cell with its adjacent passive cells. This interference is large in PEDOT:PSS pressure sensing arrays because of the low Young’s modulus (high elasticity) of the PEDOT:PSS films (Fig. 3a). Based on the schematic, we can assume that when the pressure is applied on one active cell, the surrounding passive cells are affected due to the huge deformation induced large effective influenced area of the PEDOT:PSS film, as illustrated in the cross-sectional diagram of Fig. 3c. The effective influenced area can be defined as the particular area of the PEDOT:PSS film influenced by the applied pressure. As a result, we can observe huge changes in the resistance of the adjacent and diagonal passive cells in the PEDOT:PSS pressure sensing arrays. On the other hand, the Au-NPs/PEDOT:PSS films exhibit higher Young’s modulus and hardness, which makes them more rigid and resilient as compared to the pure PEDOT:PSS films. Thus, the pressure sensing arrays fabricated with the Au-NPs/PEDOT:PSS films deform less under pressure, which contributes to the elimination of cross-talk effects. According to these observations, in addition to the changes in the piezoresistive characteristics, the mechanical properties of the PEDOT:PSS films can also be significantly modified by the incorporation of Au-NPs. The effective influenced area is much smaller in the pressure sensing arrays fabricated with the Au-NPs/PEDOT:PSS films as compared to those with the pure PEDOT:PSS films (Fig. 3b), resulting in lesser interference in the adjacent and diagonal cells, which is also illustrated in the cross-sectional diagram of Fig. 3d. The PEDOT:PSS film consists of PEDOT:PSS grains with PEDOT rich cores and PSS rich shells; the average diameters of the PEDOT:PSS grains are in the range of 30 to 50 nm^40^. In this study, Au-NPs of different sizes (5–80 nm) that were successfully incorporated in the PEDOT:PSS films act as the reinforcements that render the soft PEDOT:PSS films much more resilient to pressure. Thus, compared to the pressure sensing arrays fabricated with the PEDOT:PSS films, those fabricated with the Au-NPs/PEDOT:PSS films deform less when the pressure is applied to the active cell, which creates less interference in both the adjacent and diagonal passive cells.

**Figure 4:**
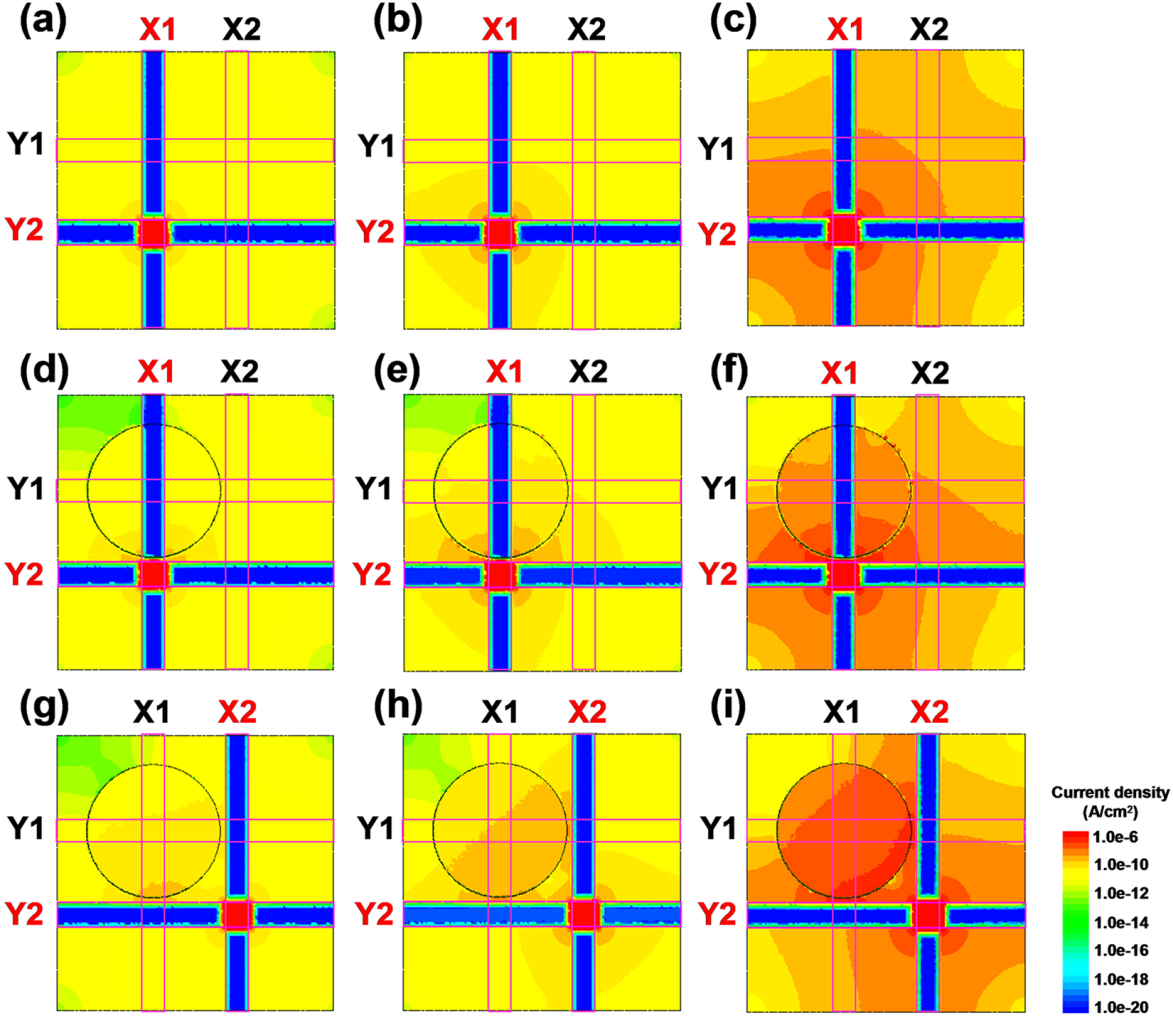
Simulated current density of the PEDOT:PSS pressure sensing 2 × 2 arrays (**a**) without and with Au-NP incorporation of (**b**) 20 and (**c**) 50 nm in diameter. The bias of bottom (Y) and top electrode (X) is 0 and 2 V respectively. For the pressure of 20 kPa applied at the active (00) cell, the distributions of current density of the adjacent (10) and diagonal (11) cells of the PEDOT:PSS pressure sensing 2 × 2 arrays without and with Au-NP incorporation of 20 and 50 nm in diameter are shown in (**d**–**i**). The tip size used for the simulation is 1.6 cm.

It is worth noting in Fig. 1f that as the size of the Au-NPs exceeds 20 nm, the pressure sensing arrays show significant cross-talk effects in spite of the high Young’s modulus and hardness of the nanocomposite films (Fig. 2). To explain this phenomenon, a current density simulation of pressure sensing arrays with PEDOT:PSS and Au-NPs/PEDOT:PSS films was performed. In our previous study, in PEDOT:PSS pressure sensors with a CPE structure, only one conducting path, i.e. the vertical conducting path from the top to the bottom of the ITO electrode, was presented^27^. The PEDOT-rich cores of the PEDOT:PSS films have a much higher intrinsic conductivity than the PSS-rich shells because PSS is a weak ionic conductor^41^. Thus, in the vertical conducting path, the PEDOT-rich domains are separated by thick PSS-lamella barriers, which enforce nearest neighbor hopping only, leading to high resistance. In the pressure sensors fabricated with the Au-NPs/PEDOT:PSS films, the nanoparticles act as conducting bridges between two PEDOT:PSS grains and boost the current density despite the presence of the weak ionic conductor PSS. Further, we studied the resistance versus pressure (*R*-*P*) characteristics of the interdigitated electrode structure (IDE) without the bottom electrode for the pressure sensors with PEDOT:PSS and Au-NPs/PEDOT:PSS films with Au-NPs of 20 and 50 nm in diameter (Supplementary Fig. S7). It is suggested that the pressure sensors with an IDE structure and without a bottom electrode have a horizontal conducting path^27^. Using the resistance values obtained from the pressure sensors with a CPE structure (Supplementary Fig. S4a) and an IDE structure without the bottom electrode (Supplementary Fig. S7c), the vertical and horizontal resistivities of the nanocomposite films without and with applied pressure of 20 kPa were extracted according to our previous work^27^ (Supplementary Table S2). In turn, the resistivity data was used to simulate the current density of the pressure sensing 2 × 2 arrays with PEDOT:PSS and Au-NPs/PEDOT:PSS films with Au-NPs of 20 and 50 nm in diameter (Fig. 4). The bias of the bottom (Y) and top (X) electrodes are 0 and 2 V respectively. The current density distributions in pressure sensing arrays with PEDOT:PSS and Au-NPs/PEDOT:PSS films with Au-NPs of 20 and 50 nm in diameter when no pressure is applied are shown in Fig. 4a–c respectively. It can be observed that the current density in the devices increases with an increase in the size of Au-NP, which leads to a decrease in the initial resistance (Supplementary Fig. S4b). The current density distributions in the adjacent (10) and diagonal (11) cells in the pressure sensing arrays with PEDOT:PSS and Au-NPs/PEDOT:PSS films with Au-NPs of 20 and 50 nm in diameter when a pressure of 20 kPa is applied at the active (00) cell are shown in Fig. 4d–i respectively. Despite being resilient to the applied pressure, it is obvious that the larger Au-NPs within the PEDOT:PSS films create highly conductive paths between the top and bottom ITO electrodes and these paths spread widely to other cells especially for the active cell, resulting in lower resistance values of the adjacent and diagonal cells. Kim *et al*. also proposed that large Au-NPs enhance the electromagnetic field around the Au-NPs under the action of an electrical bias, which can contribute to the increased current density^42^. Thus, the cross-talk effects in the pressure sensing arrays fabricated with the Au-NPs/PEDOT:PSS films become serious when the Au-NPs are larger than 20 nm in diameter (Fig. 1e). Therefore, the 20 nm Au-NPs were considered to be optimum for the incorporation in PEDOT:PSS pressure sensing arrays; the resultant arrays are nearly immune to cross-talk effects and exhibit superior piezoresistive pressure sensing characteristics. Such optimized Au-NPs/PEDOT:PSS nanocomposite films could be highly promising materials in the development of multiple-electrode pressure sensing arrays.

In summary, Au-NP incorporated PEDOT:PSS pressure sensing arrays were fabricated and their piezoresistive characteristics and cross-talk effects were investigated. The pressure sensing 2 × 2 arrays fabricated with the Au-NPs/PEDOT:PSS nanocomposite films containing Au-NPs of 20 nm in diameter (which is the optimum size) were nearly immune to cross-talk effects but at the same time exhibited excellent piezoresistive behaviors. According to the mechanical property analysis, the Au-NPs/PEDOT:PSS films present a large Young’s modulus, hardness, and plastic resistance, which make them more resilient to pressure and eliminate the interference between the active and passive cells (adjacent and diagonal) in the pressure sensing arrays. However, when the Au-NPs are larger than 20 nm, a significant cross-talk effect is observed in the pressure sensing arrays due to the high conductivity of the devices; furthermore, this observation is confirmed by a technology computer aided design (TCAD) simulation. Thus, the optimized Au-NPs/PEDOT:PSS nanocomposite films can be utilized in future high-resolution pressure sensing arrays with multiple electrodes for applications such as fingerprint identification systems.

## Methods

### Device Fabrication

Piezoresistive pressure sensing 2 × 2 arrays with Au-NPs/PEDOT:PSS films were fabricated using a 188-µm-thick flexible PET substrate covered with a 0.35-µm-thick ITO layer (Sigma-Aldrich, St. Louis, USA). ITO was used in this investigation because of its high electron density of 10^21^ cm^−3^ in the conduction band, sufficient stability in aqueous solutions for electrochemical applications^43^, and high transparency in visible light, which make it an excellent candidate for optical and solar cell applications^44^. The pressure sensing array structures are constructed using a combination of two parts – part 1 and part 2, where part 1 is the patterned ITO electrode and part 2 is the spin-coated Au-NPs/PEDOT:PSS nanocomposite film on the patterned ITO/PET substrate, as described in our previous study^27^. A 3000-µm-wide ITO electrode was patterned on the PET substrate using a standard photolithographic technique and etched using an aqua regia solution. The patterned ITO/PET substrate was cleaned with acetone and deionized (DI) water, and then treated by O_2_ plasma to make the surface of the ITO/PET film hydrophilic for the spin-coating of Au-NP incorporated PEDOT:PSS nanocomposites. A PEDOT:PSS solution (CLEVIOS P VP AI 4083) with a concentration of 1.6 wt.% and a resistivity of 785 Ω∙cm was provided by Heraeus (Leverkusen, Germany) and Au-NP solutions containing Au-NPs of different diameters (5, 10, 20, 30, 50, and 80 nm) were provided by Sigma-Aldrich (St. Louis, USA). To create the Au-NP/PEDOT:PSS solution, the Au-NP solution was mixed with the PEDOT:PSS solution at a fixed volume ratio of 1:0.3 and then shaken vigorously for 2 min in a vortex shaker to uniformly distribute Au-NPs in the PEDOT:PSS matrix. Subsequently, the mixed solution was spin coated on the O_2_ plasma treated ITO/PET substrates at a spin speed of 500 rpm and baked at 120 °C for 20 min in the ambient atmosphere. Finally, part 1 and part 2 were combined and packaged using a commercial PET material. The packaging technique was adopted to reduce the issues that arise from the not entirely intimate contact between the patterned ITO electrode of part 1 and the nanocomposite films of part 2.

### Characterization

The electrical characteristics of the fabricated piezoresistive pressure sensing devices were analyzed by using a Keithley 2450 interactive digital source meter (Keithley Instruments Inc., Cleveland, OH, USA). The samples were placed on a homemade sample holder made of rigid steel and the pressure was applied in the vertical direction using a JSV H1000 vertical stand (ALGOL Instrument Co., Ltd., Taoyuan, Taiwan) equipped with an ALGOL force gauge. To ensure an equal pressure distribution throughout the active sensing area, a quartz buffer layer of 1 cm^2^ was used. A normal pressure of 0.1–20 kPa was applied at a rate of 2 mm/sec to measure the piezoresistive characteristics of the fabricated samples. To investigate the cross-talk effects in the pressure sensing arrays, we used custom made aluminum tips with diameters in the range of 1.0 to 2.0 cm to pressurize the selected pressure sensing cell. In a standard testing, the pressure was applied on the active cell (00) and the resistance in the (01), (10), and (11) cells was measured. Both adjacent and diagonal cell characteristics were recorded to identify changes in the cross-talk effects due to Au-NP incorporation in the PEDOT:PSS films. To analyze the materials, a TEM (JEM-1230, JEOL Ltd., Japan) was used to measure the sizes of the Au-NPs; a field emission scanning electron microscope (FESEM; JSM-7500F, JEOL Ltd., Japan) was employed to confirm the incorporation of Au-NPs into the PEDOT:PSS films. Furthermore, EDS was performed to quantify the Au-NP incorporation. To understand the change in the elastic properties of the PEDOT:PSS films incorporated with Au-NPs, their Young’s modulus and hardness were measured by means of a nanoindenter (TI-900, TriboIndenter, Hysitron, USA) using a Berkovich 142.3° diamond probe at a constant indentation depth of 150 nm. The hardness values were measured from the average of six indentations on the surfaces of the PEDOT:PSS and Au-NPs/PEDOT:PSS nanocomposite films. A TCAD simulation (Sentaurus, Synopsys, Inc., U.S.A., 2013.12 Release) was also performed to understand the distribution of current density in the piezoresistive pressure sensing 2 × 2 arrays fabricated with PEDOT:PSS and Au-NPs/PEDOT:PSS nanocomposite films.

## Electronic supplementary material

Supplementary information
