# Supplementary material for: Cross-Talk Immunity of PEDOT:PSS Pressure Sensing Arrays with Gold Nanoparticle Incorporation
Source: Sci Rep. 2017 Sep 25;7:12252. doi: 10.1038/s41598-017-12420-5 (PMC5612936; doi:10.1038/s41598-017-12420-5)
Supplement: Supplementary file 1 — Supplementary information [file 41598_2017_12420_MOESM1_ESM.doc]

Supplementary Information

**Cross-Talk Immunity of PEDOT:PSS Pressure Sensing Arrays with Gold Nanoparticle Incorporation**

**Rajat Subhra Karmakar**^1,+^**,** **Yu-Jen Lu**^2,3,+^**, Yi Fu**^1^**, Kuo-Chen Wei**^2,4^**, Shun-Hsiang Chan**^5^**, Ming-Chung Wu**^5^**, Jyh-Wei Lee**^6^**, Tzu-Kang Lin**^2^**, and Jer-Chyi Wang**^1,2,7,*^

^1^Department of Electronic Engineering, Chang Gung University, Guishan Dist. 33302, Taoyuan, Taiwan

^2^Department of Neurosurgery, Chang Gung Memorial Hospital, Guishan Dist. 33305, Taoyuan, Taiwan

^3^School of Traditional Chinese Medicine, Chang Gung University, Guishan Dist. 33302, Taoyuan, Taiwan

^4^School of Medicine, Chang Gung University, Guishan Dist. 33302, Taoyuan, Taiwan

^5^Department of Chemical and Materials Engineering, Chang Gung University, Guishan Dist. 33302, Taoyuan, Taiwan

^6^Department of Materials Engineering, Ming Chi University of Technology, Taishan Dist. 24301, New Taipei City, Taiwan

^7^Department of Electronic Engineering, Ming Chi University of Technology, Taishan Dist. 24301, New Taipei City, Taiwan

^*^jcwang@mail.cgu.edu.tw

^+^these authors contributed equally to this work


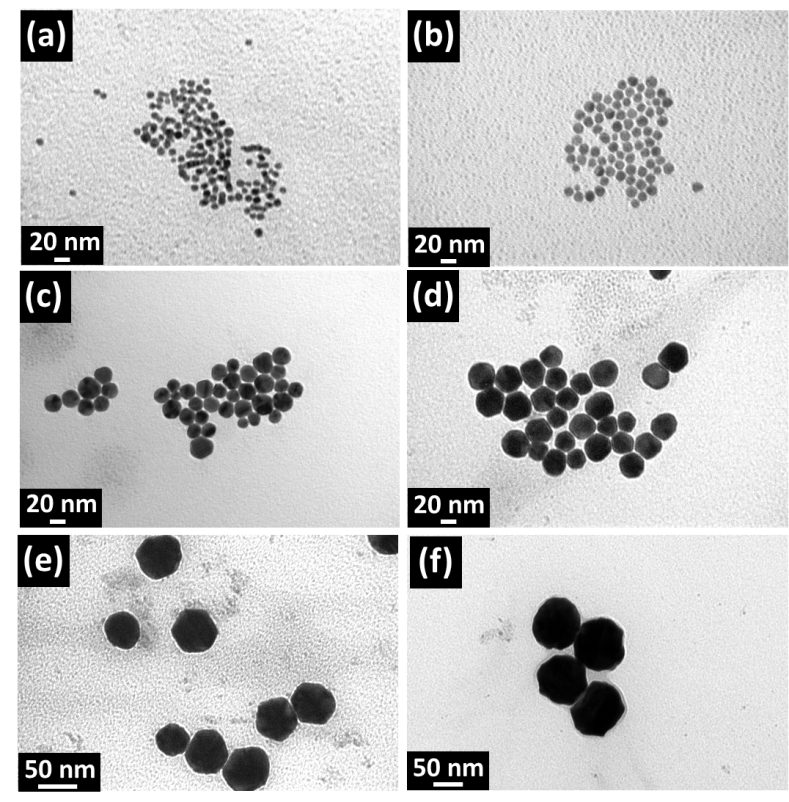


**Figure S1.** Transmission electron microscopy (TEM) images of Au-NPs denoted as (a) 5, (b) 10, (c) 20, (d) 30, (e) 50, and (f) 80 nm. The average diameters are calculated as 5.0 nm ±0.6 nm, 9.8 nm ±1.2 nm, 20.2 nm ±3.2 nm, 33.3 nm ±4.6 nm, 54.0 nm ±5.4 nm, and 85.2 nm ±9.3 nm, respectively.

**Table S1.** Summary of the Au/S ratio of the PEDOT:PSS films with different sizes of Au-NP incorporation.


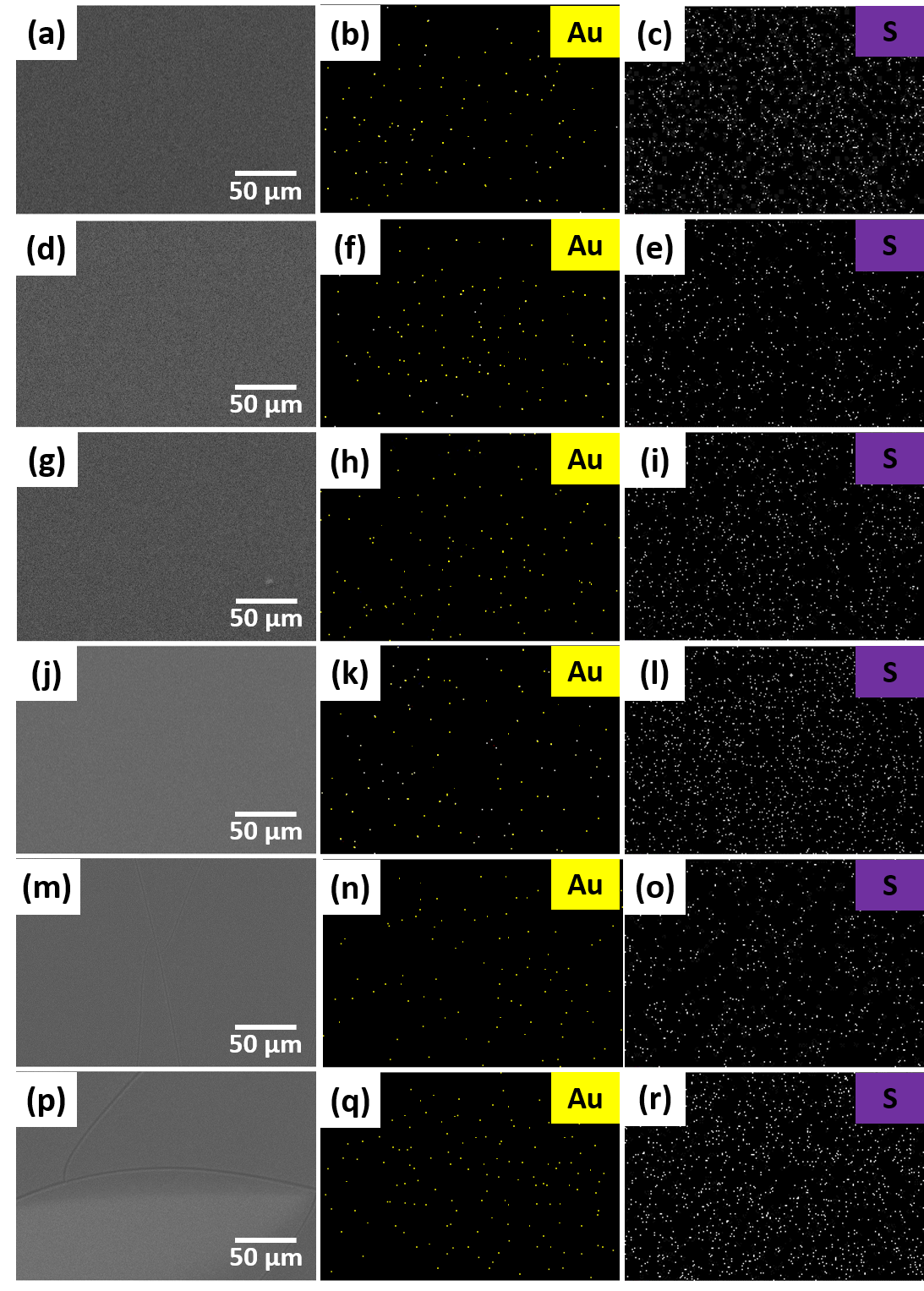


**Figure S2.** Scanning electron microscopy (SEM) images of Au-NPs/PEDOT:PSS films with Au-NPs of (a) 5, (d) 10, (g) 20, (j) 30, (m) 50, and (p) 80 nm in diameter. The energy-dispersive X-ray spectroscopy (EDS) mappings of Au atoms are shown in (b), (e), (h), (k), (n), and (q) and S atoms are shown in (c), (f), (i), (l), (o), and (r).

**Figure S3.** (a) Schematic diagram of the Au-NP incorporated PEDOT:PSS pressure sensor with cross-point electrode (CPE) structure and (b) the cross-sectional view of the pressure sensor in A-A’ direction.

**Figure S4.** (a) Resistance versus pressure (*R-P*) characteristics of the Au-NP incorporated PEDOT:PSS pressure sensors with CPE structure. (b) Piezoresistive sensitivity at the applied pressure lower than 5 kPa and the initial resistance of the pressure sensors.

**Figure S5.** (a) Reversible testing of pressure sensors with CPE structure for at least five loops. A pressure of 20 kPa applied under a 10-s holding time (*t*_h_) was used for the measurement. (b) Resistance versus time (*R-t*) behavior after the pressure release. (c) The relaxation time (*t*_r_) as a function of the size of the Au-NP incorporation in the PEDOT:PSS films. The relaxation time is defined as the waiting time required to achieve a resistance value increase of two orders of magnitude after the normal pressure is released.

**Figure S6.** Flow chart for cross-talk measurement algorithm of the PEDOT:PSS pressure sensing 2x2 arrays with Au-NP incorporation.

**Figure S7.** (a) Schematic diagram of the Au-NP incorporated PEDOT:PSS pressure sensor with inter-digitated electrode (IDE) structure and without bottom electrode and (b) the cross-sectional view of the pressure sensor in A-A’ direction. (c) *R-P* characteristics of the pressure sensor shown in (a). The Au-NPs with the sizes of 20 and 50 nm in diameter are incorporated into the PEDOT:PSS films.

**Table S2.** Summary of the calculated resistivities of the PEDOT:PSS films with Au-NP incorporation of 20 and 50 nm in diameter.
